# Polystyrene microplastics protect lettuce (*Lactuca sativa*) from the hazardous effects of Cu(OH)_2_ nanopesticides

**DOI:** 10.3389/fpls.2022.1087754

**Published:** 2022-12-08

**Authors:** Guanjie Yan, YongHao Sun, Liting Yang, Yao Zhang, Weicheng Zhang

**Affiliations:** ^1^ China‐UK‐NYNU‐RRes Joint Laboratory of Insect Biology, Henan Key Laboratory of Insect Biology in Funiu Mountain, Nanyang Normal University, Nanyang, China; ^2^ Center for Environment and Health in Water Source Area of South-to-North Water Diversion, School of Public Health, Hubei University of Medicine, Shiyan, China

**Keywords:** Cu(OH)_2_ nanopesticides, polystyrene microplastics, toxicity, lettuce, antioxidant enzymes

## Abstract

Copper-based nanopesticides are released into the environment during foliar spray application, and they could, on their own or in combination with microplastics (MPs), pose threats to environmental safety and human health. In this study, Cu(OH)_2_ nanowires greatly decreased the vigor of lettuce seeds (*p*< 0.01) and the root length of lettuce seedlings (*p*< 0.01) and significantly altered the lettuce antioxidant defence system and MDA content (*p*< 0.05). Released Cu^2+^ played a critical role in the toxicity mechanism of Cu(OH)_2_ nanowires in lettuce seedlings, as evidenced by the substantial accumulation of Cu in the seedling roots (*p*< 0.01) rather than in the leaves. Polystyrene (PS) MPs (1 mg/L) stimulated lettuce seedling growth, as shown by the (highly) significant increase in root and leaf length and in the seed vigor index (*p*< 0.01 or 0.05). Notably, PS MPs (1 mg/L) neutralized the hazardous effects of 1 mg/L Cu(OH)_2_ nanowire treatment on lettuce growth, as reflected by the vitality and root length of the seedlings returning to normal levels. The PS MPs (1 mg/L) absorbed on middle root surfaces and strongly hindered Cu accumulation in lettuce roots, which was the predominant mechanism by which PS MPs suppressed the hazardous effects of the Cu(OH)_2_ nanowires. This study strengthens the understanding of the toxicity and toxicity mechanisms of Cu(OH)_2_ nanowires with or without PS MPs in the environment.

## 1 Introduction

The nanopesticide copper(II) hydroxide (Cu(OH)_2_), as a typical engineered nanomaterial, is widely applied in agriculture for the purpose of plant growth due to its excellent antimicrobial and antifungal properties (Li et al., 2019; Bindra and Singh, 2021; Konappa et al., 2021). Inevitably, a large amount of Cu(OH)_2_ nanopesticides will be released into the environment when they are sprayed on plants (Conway, 2015), and thus, Cu(OH)_2_ nanopesticides could pose serious threats to environmental safety and human health (Nair and Chung, 2015). Correspondingly, the environmental fate, transport, bioavailability and toxicity of Cu(OH)_2_ nanopesticides are now being extensively investigated to elucidate their potentially hazardous effects on different plant species (Zuverza-Mena et al., 2015; Du et al., 2018). For example, studies have shown that Cu(OH)_2_ nanopesticides can induce oxidative stress, defence responses and enhanced Cu intake in lettuce (*Lactuca sativa*) and basil (*Ocimum basilicum*); can cause a reduction in antioxidant and defence-associated metabolites in spinach (*Spinacia oleracea*); and can bring about metabolic reprogramming in both cucumber (*Cucumis sativus*) and corn (*Zea mays*) (Zhao et al., 2016a; Zhao et al., 2016b; Zhao et al., 2017c; Tan et al., 2018). In addition to exhibiting effects on plants, Cu(OH)_2_ nanopesticides have been found to have toxic effects on other organisms, such as *Leptocheirus plumulosus*, zebrafish, and microbes, as well as on human hepatocellular carcinoma cells (Aksakal and Sisman, 2020; Vignardi et al., 2020; Zhang et al., 2020). Notably, the environmental and human health risks due to contamination of Cu(OH)_2_ nanopesticides in water and residues on food products are still poorly understood. Moreover, MPs have been detected extensively in soil environments, organisms, and even humans (de Souza Machado et al., 2018; Hermsen et al., 2018; Ragusa et al., 2021; Song et al., 2021; Leslie et al., 2022). However, the adverse effects of coexisting Cu(OH)_2_ nanopesticides and microplastics (MPs) on plants have not been formally investigated, although the adverse effects of Cu^2+^ in concert with MPs on plant seedling growth have attracted attention recently (Zong et al., 2021; Zhou et al., 2022).

Interestingly, MPs have shown two different effects on plant growth: inhibition and stimulation. From the inhibition perspective, MPs showed an adverse effect on seedling growth and the antioxidant system of wheat (*Triticum aestivum*) (Liu et al., 2021), on growth, photosynthesis and essential elements in *Cucurbita pepo* (Colzi et al., 2022), and on rice seedlings (Dong et al., 2020). From the stimulation perspective, MPs can stimulate plant growth, as expressed by root length increases (Lian et al., 2020; Lozano and Rillig, 2020; Liu et al., 2022; Zeb et al., 2022). This plant growth stimulation could be explained by the MPs increasing carbon and nitrogen levels in the plants (Lian et al., 2020; Liu et al., 2022) and improving the aeration and penetration of roots (Lozano and Rillig, 2020). Due to concerted efforts by scientists, the potential effects of MPs on the environment have been determined, although high concentrations of MPs were applied in only some of these studies. Nevertheless, the effects of MPs on plant growth at environmental concentrations (e.g., 1 mg/L) have not been investigated. Given that both MPs and other contaminants can coexist in the environment for a long time, MPs can serve as vectors for other contaminants, such as Cu (Zong et al., 2021; Zhou et al., 2022), Cd (Zong et al., 2021; Zeb et al., 2022), Ag^+^ (Sun et al., 2020), As(III) (Dong et al., 2020), phenanthrene (Liu et al., 2021), oxytetracycline (Bao et al., 2021), and nanomaterials (Li et al., 2020; Yang et al., 2021; Tong et al., 2022a; Tong et al., 2022b), and can modify the toxicity of these environmental contaminants to biota (Fries et al., 2013; Pacheco et al., 2018; Li et al., 2020; Yang et al., 2021; Zhang et al., 2021; Tong et al., 2022a; Tong et al., 2022b). In principle, MPs likely coexist with Cu(OH)_2_ nanopesticides in the environment and induce coupled effects on plant growth, due to they are share the entry path into environment and share the major sink a long time in environment (Rajput et al., 2020; Wang et al., 2021). Accordingly, there are synergistic or antagonistic effects of Cu(OH)2 nanopesticides and MPs on plant growth, which are consistent with the inhibitory or stimulatory effects of MPs on plant growth. Nevertheless, it is still unclear whether environmental concentrations of polystyrene (PS) MPs drive the bioavailability and toxicity of Cu(OH)_2_ nanopesticides in the context of plant seed germination or seedling growth.

To bridge these gaps, Lettuce (*Lactuca sativa*), which is a model plant and is widely applied in toxicity assays (Zhao et al., 2016a; Zhao et al., 2016b; Gao et al., 2021; Zeb et al., 2022), was applied in this study to explore the physiological and biological effects of Cu(OH)_2_ nanopesticides and/or MPs on plants. Therefore, we hypothesized that Cu(OH)_2_ nanowires could induce hazardous effects on *L. sativa* seed germination and seedling growth after Cu(OH)_2_ nanopesticides enter the environment. Furthermore, we proposed that PS MPs that have been widely detected in realistic environments (Zhang et al., 2018; Ding et al., 2021) possibly stimulate lettuce growth at an environmental concentration (1 mg/L) and thus protect lettuce from the toxicity of Cu(OH)_2_ nanopesticides. Accordingly, this experiment aimed to (i) determine the hazardous effects of Cu(OH)_2_ nanowires on seed germination and seedling growth of lettuce and carry out a biological analysis, and (ii) explore the effects of PS MPs (1 mg/L) on stimulating lettuce seedling growth and suppressing Cu(OH)_2_ nanowire toxicity to lettuce.

## 2 Materials and methods

### 2.1 Materials

PS MPs (0.1 µm) and fluorescent PS MPs were purchased from Tianjin Baseline Chromtech Research Centre, China. Cu(OH)_2_ nanowires (Product No. CuOH-NW-40) were obtained from Beijing Dk Nano Technology Co., Ltd. Scanning electron microscopy (SEM, GeminiSEM 500, Zeiss) was employed to determine the sizes and morphologies of the PS MPs and Cu(OH)_2_ nanowires. The hydrodynamic diameters and zeta potentials of the PS MPs and/or Cu(OH)_2_ nanowires were determined with a Zetasizer Nano instrument (1000S, Malvern Instruments, Ltd., UK). Lettuce (*Lactuca sativa*) seeds were purchased from Nanyang Seed Inc. (Henan, China). Peroxidase (POD), superoxide dismutase (SOD), malondialdehyde (MDA) and catalase (CAT) activity assay kits were purchased from Nanjing Jiancheng Bioengineering Institute, Nanjing, China.

### 2.2 Lettuce culture and Cu and/or PS MPs exposure

The lettuce seeds were surface disinfected in hydrogen peroxide (3%, v/v) for 30 min with continuous stirring, rinsed 3 times with deionized water (DI water), and immersed in DI water for 24 h. The seeds were randomly divided into eight different groups (100 seeds), including a control group (CK), a 1 mg/L Cu(OH)_2_ nanowire group, a 10 mg/L Cu(OH)_2_ nanowire group, a 10 mg/L Cu^2+^ group, a PS (1 mg/L) group, a PS (1 mg/L) + 1 mg/L Cu(OH)_2_ nanowire group, a PS (1 mg/L) + 10 mg/L Cu(OH)_2_ nanowire group, and a PS (1 mg/L) + 10 mg/L Cu^2+^ group. The 10 mg of Cu(OH)_2_ nanowire were added into a 100 glass measuring flask and sonicated on an ultrasonic water bath (0.5 min) (ready-to-use). Certain of Cu(OH)_2_ nanowire/Cu^2+^(CuSO_4_) were added to certain of 100 mg/L of PS MPs solution or DI water to obtain the above different exposure groups. Four replicates for these groups were performed, and 100 seeds were placed in each glass Petri dish (⌀ 90 mm). Depending on the treatment group to which the seeds were randomly assigned, the seeds were incubated with 7 mL of the assigned Cu(OH)_2_ nanowires or Cu^2+^ with or without PS solution and were kept in the dark at 25°C for seed germination.

### 2.3 Germination, lettuce seedling, biomass, oxidative stress and chlorophyll measurements

The germination rate was observed and recorded for three days. Accordingly, the germination rate, germination index, vigor index and germination energy of the seeds were calculated by equations (1) to (4). The biomass of the lettuce seedlings was measured, as well as the root length and shoot height, and the fresh weights of shoots and roots were recorded.


(1) Germination rate (GR,%) = Seed germination numberTotal number of tested seeds x 100

(2) $$\text{Germination index }\left(\text{GI}\right)\text{ = }\sum^{}\text{ (}\frac{{\text{G}}_{\text{t}}}{{\text{D}}_{\text{t}}})$$

3) Vigor index (VI) = GI x fresh weight of seedlin

(4) Germination energy (GE,%) = Seed germination number in 3 daysTotal number of tested seeds x; 100

Where GR and GI represent the germination rate and germination index, respectively. Gt represents the germination number at t days, and Dt represents the corresponding germination time (days). VI and GE represent the vigor index and germination energy, respectively.

Antioxidant enzyme levels, including SOD, CAT and POD activity levels and MDA levels, were measured with commercial assay kits obtained from Nanjing Jiancheng Bioengineering Research Institute during the seed germination (3 d) and seedling growth (7 d) stages. The measurement processes for the antioxidant enzyme activities were in accordance with the manufacturer’s instructions. Chlorophyll a (Chl a) and chlorophyll b (Chl b) were measured at the seedling growth stage (7 d). A specific weight (m) of freeze-dried cotyledons of lettuce samples was ground in a mortar with quartz sand and then extracted with 80% acetone to a specific volume (V). The obtained supernatant was centrifuged (5000 r/min for 10 min at 4 °C), and the absorbance was measured at 663 and 645 nm with UV−Vis spectrometry. Finally, Chl a and Chl b were calculated according to equations (5) and (6), respectively.


(5) $$\text{Chl a (mg/g)= (12}.7 *\;{\text{OD}}_{663}\;-2.6\;*\;{\text{OD}}_{645)}\;*\;V/m$$

(6) $$\text{Chl b (mg/g)= (22}.9 *\;{\text{OD}}_{645}\;-\;4.7\;*\;{\text{OD}}_{663)}\;*\;V/m$$

Where m is the specific weight of the lettuce cotyledon sample and V is the specific volume created by adding 80% acetone to the ground cotyledons.

### 2.4 Uptake of PS MPs by lettuce seeds and seedlings

Surface-disinfected lettuce seeds were immersed in DI water for 24 h, after which 100 of these seeds were selected and exposed to 1 mg/L fluorescent PS MPs. The seeds were cultured with fluorescent PS MPs (total volume of 7 mL) for seed germination. After 3 d of exposure in the dark at 25°C, the lettuce seed and seedling samples were washed three times with DI water to exclude fluorescent PS MPs adsorbed on the root surface. Then, the uptake of fluorescent PS MPs by the lettuce seeds and seedlings was examined using fluorescence microscopy (Nikon SMZ1500, Japan) with a photomicrography system (Nikon DS-Fi1c, Japan). The excitation wavelength of the fluorescence microscope was 488 nm, and the emission wavelength was 518 nm.

### 2.5 Cu accumulation in lettuce seeds and seedlings

Ten of lettuce seeds (at 3 d) and seedlings (at 7 d) samples were taken and washed with EDTA-2Na (0.1 mM; pH 6.0) to exclude extracellular Cu^2+^ or Cu(OH)_2_ on the seed and seedling surfaces. Then, the sample tissues were washed three times with DI water and oven-dried to constant weight for digestion (by adding 4 mL of a mixture (1:3) of H_2_O_2_ and HNO_3_ and heating at 80 °C for 2 h and then at 160°C for 4 h) and Cu accumulation quantification (by inductively coupled plasma−mass spectrometry).

### 2.6 Statistical analysis

The experimental data were for four replicates were analyzed with SigmaPlot 12.5. The data are presented as the means ± standard deviations. These data were normally distributed and were evaluated with a *t test* and/or one-way ANOVA to explore any significant differences between treatment groups. Significant differences are marked with asterisks (* (*p*< 0.05) and ** (*p*< 0.01), denoting statistically significant and highly statistically significant differences, respectively).

## 3 Results and discussion

### 3.1 Characterization of Cu(OH)_2_ nanowires and PS MPs

**Figure 1:**
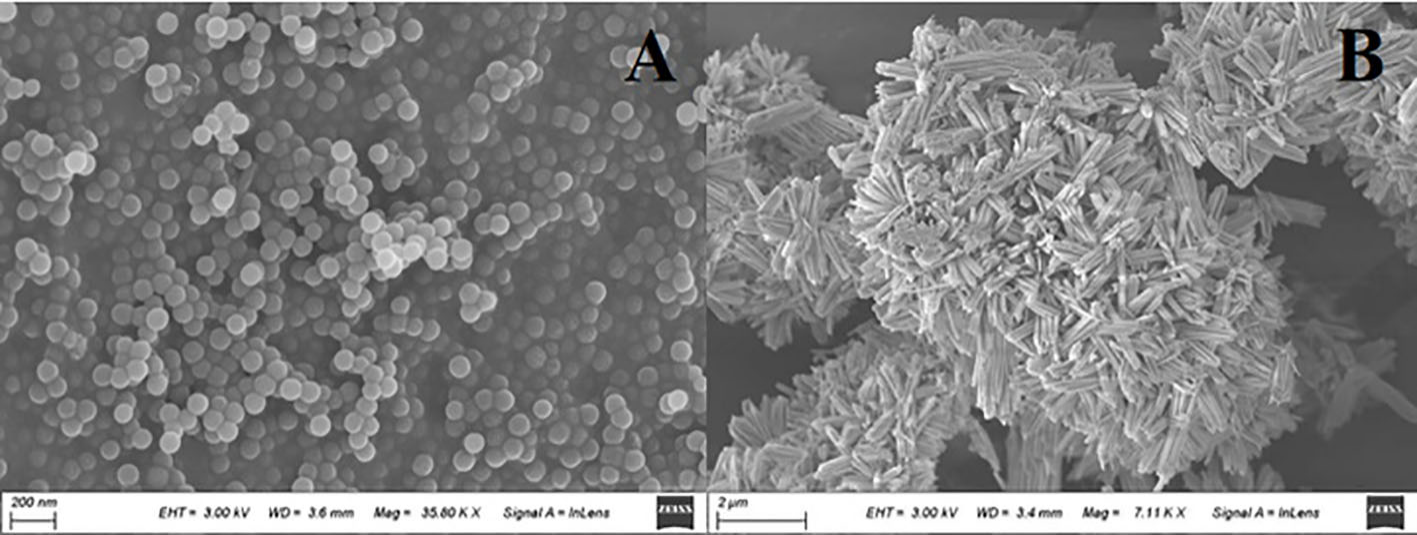
SEM images of the PS MPs **(A)** and Cu(OH)_2_ nanowires **(B)**.

The PS MPs exhibited a spherical shape with quite a smooth surface and had an average size of ~ 100 nm (**Figure 1A**). They were stable in ultrapure water (1 mg/L) according to their average hydrodynamic diameter of 182.46 (± 74.34) and zeta potential of –13.33 (± 0.61) mV. The Cu(OH)_2_ nanowires exhibited a typical wire shape with a nanosized diameter (40-80 nm) and a microsized length (1-2 µm) (**Figure 1B**). According to the dynamic light scattering (DLS) results, the Cu(OH)_2_ nanowires tended to aggregate slightly in ultrapure water (1 mg/L), based on their average hydrodynamic diameter of 1584.97 (± 674.29) nm and on their average zeta potential of –21.58 (± 1.81) mV. Interestingly, the Cu(OH)_2_ nanowires (1 mg/L) with PS MPs (1 mg/L) presented a large hydrodynamic diameter of 2365.25 (± 856.37) nm and a low zeta potential of –35.12 (± 5.47) mV and tended to aggregate in ultrapure water.

### 3.2 Effects of Cu(OH)_2_ nanowires and Cu^2+^ on lettuce seed germination and seedling growth

**Figure 2:**
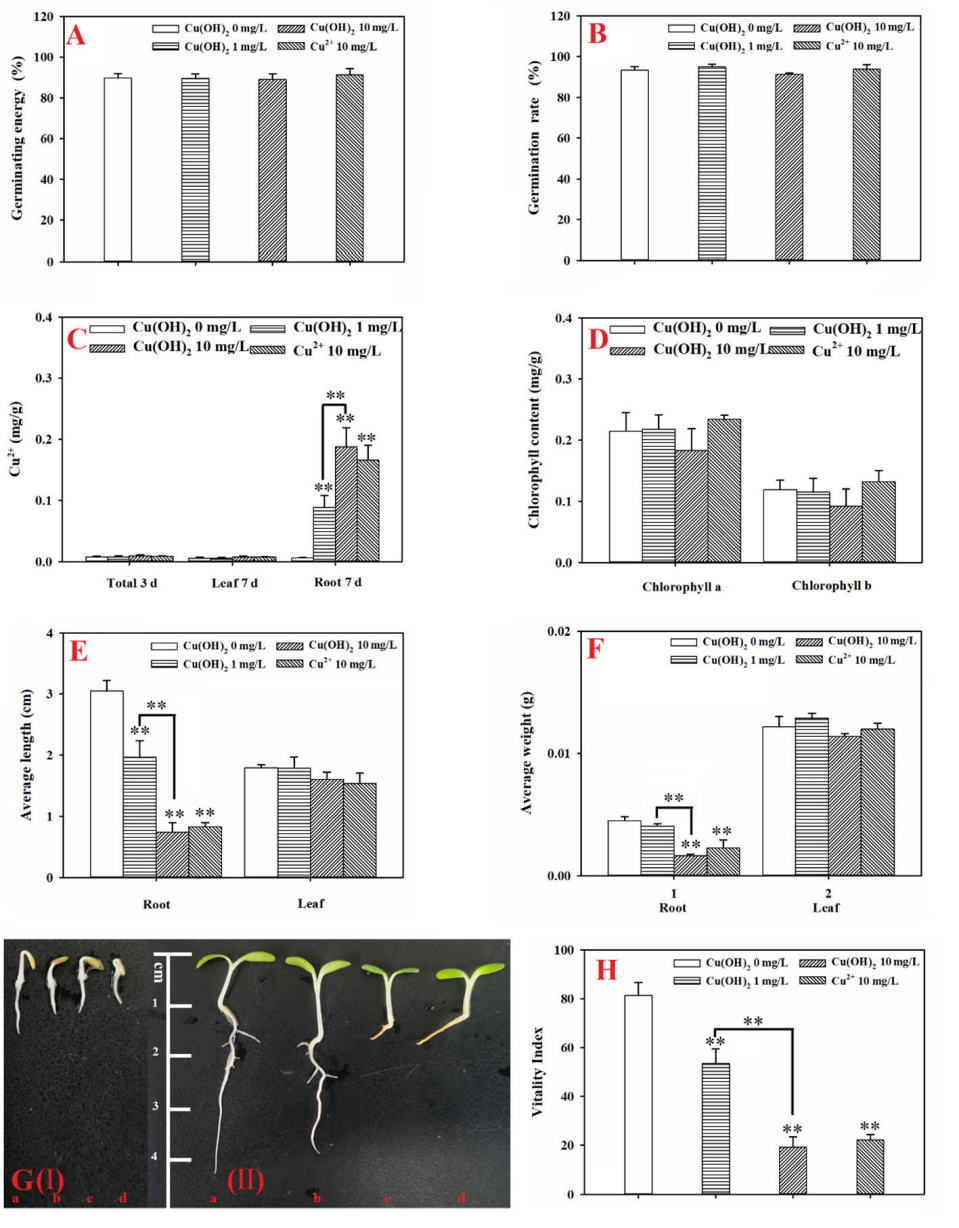
**(A)**: Germination energy of lettuce seeds under different treatments; **(B)**: germination rate of lettuce seeds under different treatments; **(C)**: accumulation of Cu in lettuce seeds (3 d) and seedling leaves and roots (7 d) under different treatments; **(D)**: chlorophyll content in seedling leaves (7 d) under different treatments; **(E)**: average length of roots and leaves (7 d) under different treatments; **(F)**: average weight of roots and leaves (7 d) under different treatments; **(G)**: lettuce seed germination at 3 days (I) and lettuce seedling growth at 7 days (II). The letters a, b, c, and d denote the control, 1 mg/L Cu(OH)_2_, 10 mg/L Cu(OH)_2_, and 10 mg/L Cu^2+^ treatment groups, respectively; and **(H)** vigor index of lettuce seeds (7 d) under different treatments. The data are presented as the mean ± SD from at least four replicates. ** means extremely significant difference.

**Figure 3:**
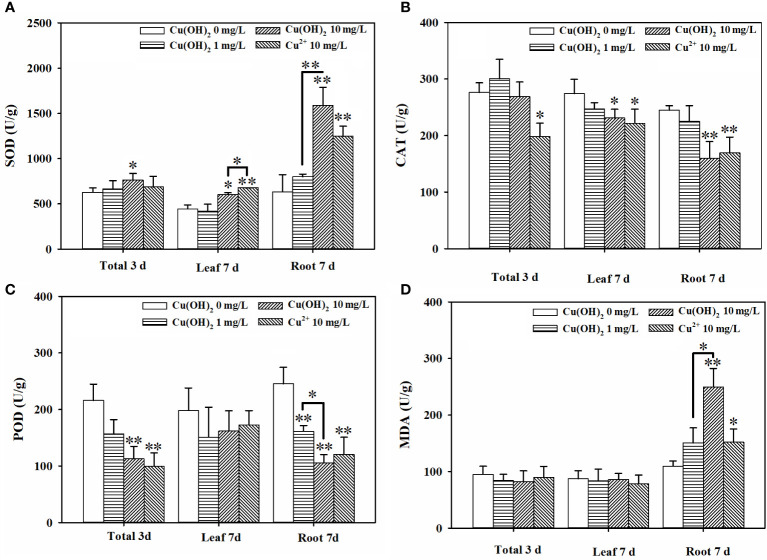
Effect of Cu(OH)_2_ nanowire or Cu^2+^ stress on the activities of SOD **(A)**, CAT **(B)** and POD **(C)** and level of MDA **(D)** in lettuce seed (3d) and in lettuce seedling leaves and roots (7d). The data are shown as the mean ± SD from the four replicates and were analyzed with a *t test* to explore any significant differences between treatment groups; asterisks * (*p*< 0.05) and ** (*p*< 0.01) denote statistical significance and high statistical significance, respectively.

Based on the seed germination period (3 d) data, the Cu(OH)_2_ nanowires and Cu^2+^ stress had no significant effect on the germination energy or the germination rate of the lettuce seeds (*p* > 0.05; **Figures 2A, B**). It is possible that the seed husks protected the lettuce seeds from the Cu(OH)_2_ nanowires and Cu^2+^ stress during seed germination. Indeed, Cu ions hardly accumulated during the 3 d seed germination period (*p* > 0.05; **Figure 2C**). Nevertheless, compared with the radicle length of germinants in the water treatment groups, the radicle length was visibly shorter in the germinants of the Cu(OH)_2_ nanowire and Cu^2+^ treatment groups (**Figures 2G**(I)). Moreover, the antioxidant defence system and the MDA content in seedlings were significantly modified under Cu stress, as reflected by the significant increase in SOD activity and the (highly) significant decrease in CAT and POD activities in the 10 mg/L Cu(OH)_2_ nanowire and Cu^2+^ treatments (*p*< 0.01 or 0.05; **Figures 3A–C**), respectively. It has been proposed that the release of Cu^2+^ may be the underlying mechanism by which Cu(OH)_2_ nanowires induce toxic effects in plants (Zhao et al., 2017c). In this study, the 10 mg/L Cu^2+^ stress treatment induced adverse effects comparable to those induced by the 10 mg/L Cu(OH)_2_ nanowire stress treatment, suggesting that released Cu ions played a critical role in the toxicity effects induced by Cu(OH)_2_ nanowires.

Based on the seedling growth period (7 d) data, the Cu(OH)_2_ nanowire and Cu^2+^ treatments (highly) significantly inhibited the average root length (*p*< 0.01; **Figure 2E**) and weight (*p*< 0.05 or *p*< 0.01; **Figure 2F**). Correspondingly, the Cu(OH)_2_ nanowire and Cu^2+^ treatments greatly decreased the vitality of the lettuce seedlings (*p*< 0.01; **Figure 2H**). This inhibition by the Cu(OH)_2_ nanowires showed a dose‐dependent effect (**Figures 2E, F, H**), which has implications for the health risk posed by Cu(OH)_2_ nanowires in the environment. Notably, the 10 mg/L Cu^2+^ and Cu(OH)_2_ nanowire treatments displayed similar effects, (highly) significantly inhibiting the average root length and weight, as well as adversely affecting the vitality of the lettuce seedlings, which again emphasizes the important role of Cu^2+^ release in the hazardous effects induced by Cu(OH)_2_ nanowires. Additionally, the antioxidant defence system and MDA content were significantly modified under Cu stress at 7 d of lettuce seedling growth. For example, compared with the SOD activity and MDA levels in seedling roots or leaves from the water treatment groups, those in the 10 mg/L Cu(OH)_2_ nanowire or Cu^2+^ treatments were (highly) significantly increased (*p*< 0.01 or 0.05; **Figures 3A, C**). Furthermore, compared to the CAT and POD activities in seedling roots or leaves in the water treatment groups, those in the 10 mg/L Cu(OH)_2_ nanowire or Cu^2+^ treatment groups were (highly) significantly decreased (*p*< 0.01 or 0.05; **Figures 3B, C**). Compared with the effect of Cu stress on seedling roots, Cu stress did not induce serious adverse effects on seedling leaves, as reflected by the chlorophyll a and b levels (*p* > 0.05; **Figure 2D**), leaf length (*p* > 0.05; **Figure 2E**) and leaf weight (*p* > 0.05; **Figure 2F**), which were not significantly modified under Cu stress. In contrast, other studies have detected biomass and chlorophyll content decrease, antioxidant-related enzyme modifications, metabolite profile alterations and antioxidant or defence-associated metabolite reductions in different plant leaves after Cu(OH)_2_ nanopesticide or Cu^2+^ exposure through foliar spraying (Zhao et al., 2017a; Zhao et al., 2017b; Zhao et al., 2017c). It seems that the hazardous effects Cu stress induces in plants are strongly dependent on the exposure pathways. Indeed, in this study, the seedling roots were directly exposed to Cu(OH)_2_ nanowires or Cu^2+^ solution, and thus, Cu significantly accumulated in seedling roots (*p*< 0.05; **Figure 2C**) rather than in seedling leaves. Notably, it has been proposed that the free Cu^2+^ released from Cu(OH)_2_ nanopesticides may be a nonnegligible toxicity mechanism of the nanopesticides. Comparably, Cu was shown to predominantly accumulate in plant leaves but not in roots when a Cu(OH)_2_ nanopesticide was applied through foliar spraying (Zhao et al., 2016b; Tan et al., 2018).

### 3.3 PS MPs (1 mg/L) stimulate lettuce seedling growth

**Figure 4:**
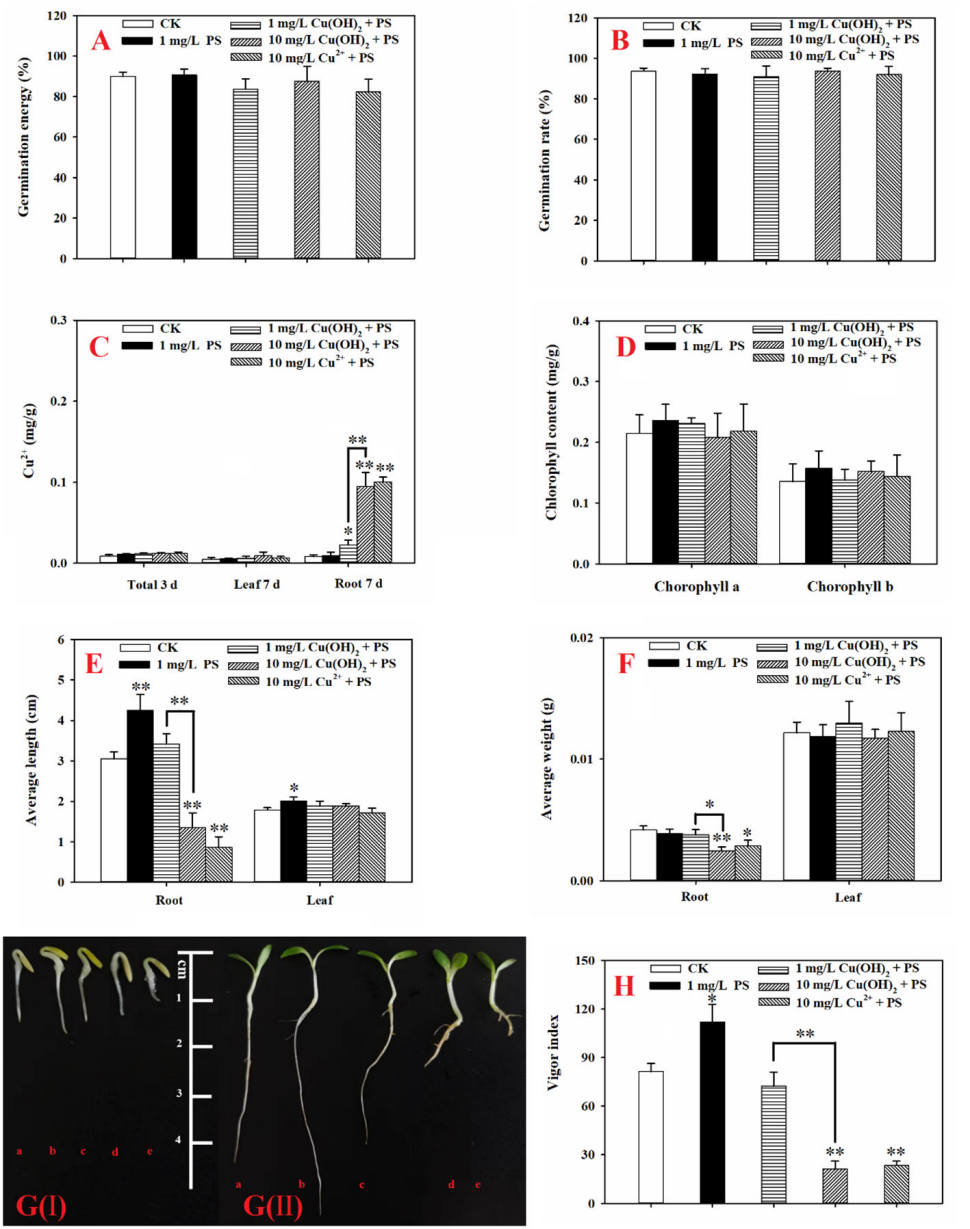
**(A)**: Germination energy of lettuce seeds under different treatments; **(B)**: germination rates of lettuce seeds under different treatments; **(C)**: accumulation of Cu in lettuce seeds (3 d) and in seedling leaves and roots (7 d) under different treatments; **(D)**: chlorophyll content in seedling leaves (7 d) under different treatments; **(E)**: average length of roots and leaves (7 d) under different treatments; **(F)**: average weights of roots and leaves (7 d) under different treatments; **(G)**: lettuce seed germination at 3 d (I) and lettuce seedling growth at 7 d (II). The letters a, b, c, and d denote the control, 1 mg/L Cu(OH)_2_, 10 mg/L Cu(OH)_2_, and 10 mg/L Cu^2+^ treatment groups, respectively; and **(H)**: vigor index of lettuce seedlings (7 d) under different treatments. The data are shown as the mean ± SD from the four replicates and were analyzed with a *t test* to explore any significant differences between treatment groups; the asterisks * (*p*< 0.05) and ** (*p*< 0.01) denote statistical significance and high statistical significance, respectively.

**Figure 5:**
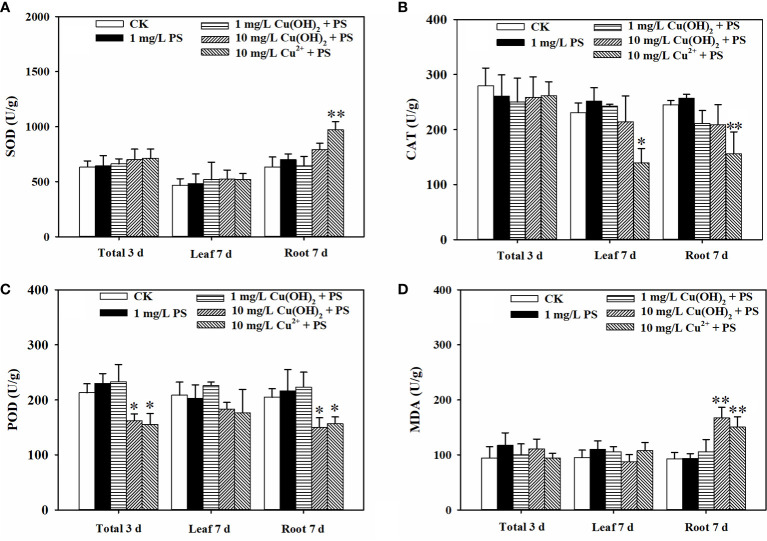
Effect of 1 mg/L PS MPs with or without Cu(OH)_2_ nanowire or Cu^2+^ stress on the activities of SOD **(A)**, CAT **(B)** and POD **(C)** and the MDA levels **(D)** in lettuce seeds (3d) and in lettuce seedling leaves and roots (7d). The data were from four replicates and were analyzed with a *t test* to explore any significant differences between treatment groups; the asterisks * (*p* < 0.05) and ** (*p* < 0.01) denote statistical significance and high statistical significance, respectively.

**Figure 6:**
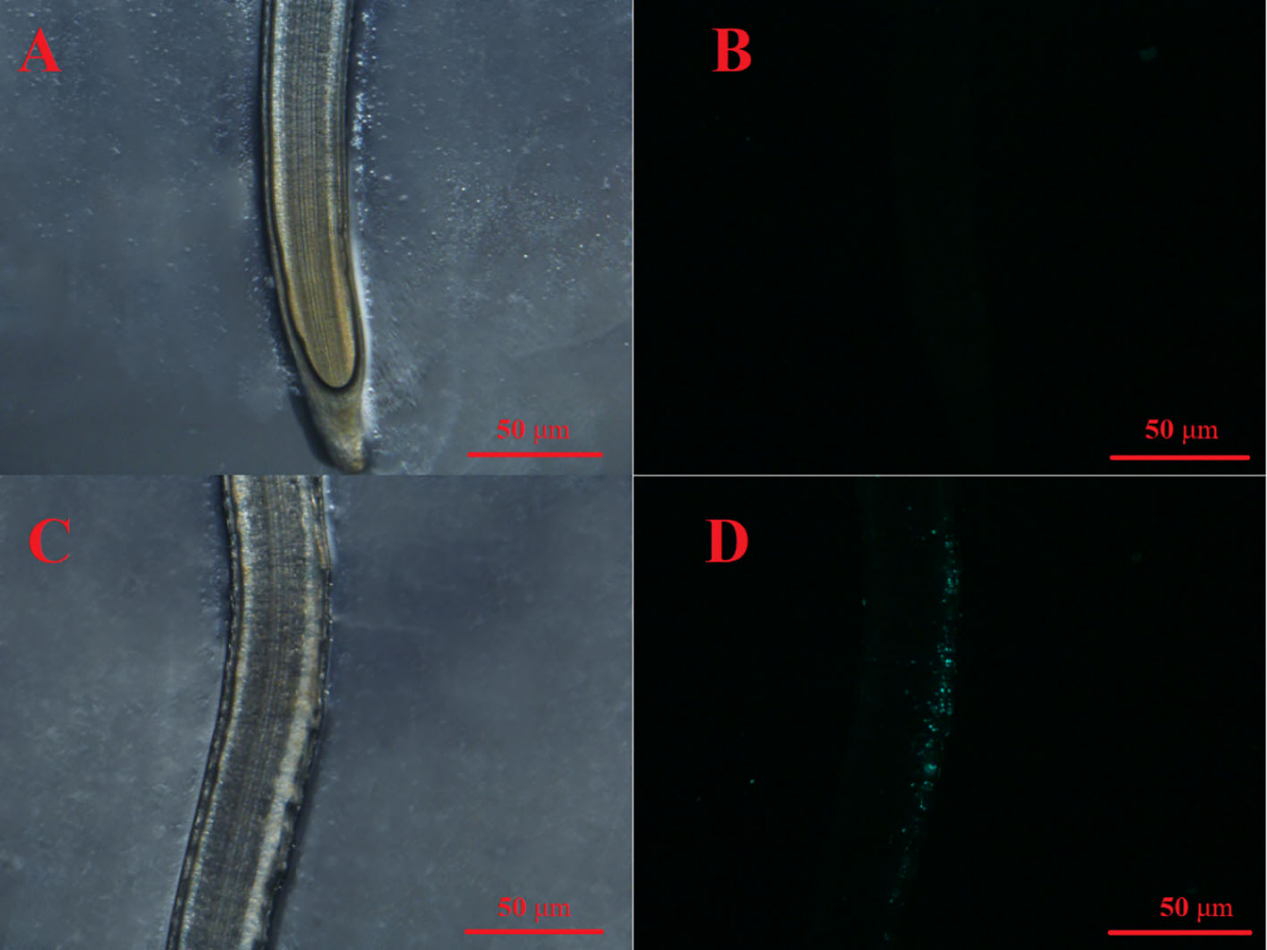
Seedling roots with 1 mg/L fluorescent PS MPs (0.1 μm) after 3 d of exposure. **(A)**: Root tip under normal light. **(B)**: Fluorescent signal in the same root tip shown in **(A, C)** Middle segment of a root under normal light. **(D)**: Fluorescent signal in the middle segment of the same root shown in **(C)**.

PS MPs at an environmental concentration of 1 mg/L stimulated the seed germination and seedling growth of lettuce. To elaborate, lettuce seed germination was noticeably increased by the 1 mg/L PS MPs treatment [**Figure 4G**(I)], although the germination energy and rate were hardly affected (*p* > 0.05; **Figures 4A, B**). Moreover, the PS MPs treatment (highly) significantly increased the root and leaf length of the lettuce seedlings (**Figure 4E**) and significantly increased the seed vigor index (*p*< 0.05; **Figure 4H**). Similarly, a significant increase in root length in response to PS MPs (0.01 to 1 mg/L) and polyester microfiber treatments has also been observed in other plants (Lian et al., 2020; Lozano and Rillig, 2020; Liu et al., 2022; Zeb et al., 2022). The root length enhancements in response to MPs treatment could be interpreted as both PS MPs and cellulose cell walls are highly hydrophobic which makes PS MPs adsorb on the surface of the roots (Nel et al., 2009; Dovidat et al., 2019), increasing carbon and nitrogen levels in the plant (Lian et al., 2020; Liu et al., 2022) and improving the aeration and penetration of roots (Lozano and Rillig, 2020). In this study, the PS MPs had no significant effect on the levels of chlorophyll a or b in lettuce leaves (*p* > 0.05; **Figure 4D**). In contrast, PE MPs and polyester microfibers have been found to increase the amount of chlorophyll in other plants (Wang et al., 2020; Liu et al., 2021; Zeb et al., 2022). Similarly, 1% PE MPs promoted carotenoids to reach the highest value in wheat leaves (Liu et al., 2021) and increased the total chlorophyll concentration in maize leaves (Wang et al., 2020). Accordingly, photosynthesis system stimulation by PE MPs could partially explain the plant growth increase (e.g., root length). In addition, the activities of SOD, CAT, and POD and the level of MDA in the lettuce leaves was hardly affected by the PS MPs (**Figure 5**), implying that the root, rather than the leaf, was the target organ of the PS MPs in this research. Furthermore, the adsorption of PS MPs on the middle root surface, but not on the root tips, was confirmed, as shown in **Figure 6**.This could partially explain why the PS MPs tended to affect the lettuce during the seedling growth stage rather than during the seed germination period (**Figures 4A, B**). However, significant PE MPs stimulation of SOD, CAT and POD activities (*P*< 0.05) and bottom-to-top transportation of PS MPs have been confirmed in wheat roots (Lian et al., 2020; Liu et al., 2021). It is possible that the different MPs types (PS vs. PE), sizes (nm vs. μm) and concentrations (low vs. high) are the causes of these inconsistent results.

### 3.4 Combined effects of PS MPs (1 mg/L) and Cu(OH)_2_ nanowires or Cu^2+^ on lettuce seed germination and seedling growth

In this study, the 1 mg/L PS MPs treatment significantly suppressed the hazardous effects induced by Cu(OH)_2_ nanowires or Cu^2+^ stress. Based on the seed germination period data (3 d), the PS MPs treatment helped SOD and CAT activities return to the control level (*p* > 0.05), and these activities were the dominant mechanisms for protection of lettuce seed germination against Cu(OH)_2_ nanowire or Cu^2+^ stress (as mentioned in Section 3.2). Furthermore, when PS MPs were present, the highly decreased vigor index that was induced by the 1 mg/L Cu(OH)_2_ nanowire treatment was reversed, and the vigor index returned to the control level (*p* > 0.05; **Figure 4H**). However, PS MPs treatment hardly affected the germination energy, the germination rate (**Figures 4A, B**), and Cu accumulation of the lettuce seeds (**Figure 4C**) in the Cu(OH)_2_ nanowire or Cu^2+^ treatments.

Based on the seedling growth data (7 d), the decrease in lettuce seedling root length caused by the 1 mg/L Cu(OH)_2_ nanowire treatment was significantly restrained by PS MPs treatment, as shown in **Figures 4E, G**(II). Moreover, the PS MP treatment partially suppressed the hazardous effects of the 10 mg/L Cu(OH)_2_ nanowire or Cu^2+^ treatments on seedling root growth, although the average seedling root length in these treatment groups was still much shorter than that in the water treatment groups (*p*< 0.01; **Figure 4E**). As mentioned in Section 3.2, the dominant mechanisms of the decrease in seedling root length caused by Cu stresses were alterations of the antioxidant defence system, changes in MDA content and the accumulation of Cu. Compared with the SOD, CAT and POD activities in seedling roots or leaves in the single 10 mg/L Cu(OH)_2_ nanowire or Cu^2+^ treatment groups, respectively, the activities in the treatment groups with 10 mg/L Cu(OH)_2_ nanowires and Cu^2+^ with PS MPs were (highly) significantly decreased. Furthermore, the MDA level in the 10 mg/L Cu(OH)_2_ nanowire treatment group was significantly decreased by the presence of PS MPs, although it was still much higher than that in the control (**Figure 5D**). Compared to the overall Cu levels in the single Cu(OH)_2_ nanowire or Cu^2+^ treatment groups, the Cu level in seedling roots was also highly significantly decreased (*P*< 0.01) by the 1 mg/L PS MPs treatment. For example, the Cu concentration from the Cu(OH)_2_ nanowire (1 mg/L) treatment decreased from 0.093 (± 0.017) to 0.023 (± 0.008) mg/g with PS MPs + Cu(OH)_2_ nanowire (1 mg/L) treatment, and thus, Cu^2+^ bioavailability was decreased by PS MPs treatment in this study. Furthermore, the PS MPs were likely adsorbed on the surface of the middle root section (**Figures 6C, D**) rather than on the root tips (**Figures 6A, B**) and in turn decreased Cu^2+^ bioavailability. Notably, the uptake of nanosized MPs into plant roots has been determined previously (Jiang et al., 2019; Lian et al., 2020; Liu et al., 2021), and it has been shown that PS MPs vehicle effects on the biotic uptake of heavy metals (such as Zn^2+^ and Ag^+^) are possible (Tong et al., 2022a; Tong et al., 2022b). However, in this study, the PS MPs bound to the lettuce root surface (**Figure 6**) rather than becoming embedded in the root. Thus, PS MPs treatment did not promote Cu accumulation in lettuce roots or leaves during the seedling growth period (**Figure 4C**). In contrast, the PS MPs might have prevented the Cu(OH)_2_ nanowires or Cu^2+^ from entering the lettuce by binding to the lettuce root surface.

The protective effects of the MPs treatment on other plants have also been previously confirmed. For example, the presence of PE MPs reduced the accumulation of phenanthrene in wheat roots and leaves (Liu et al., 2021), decreased the dibutyl phthalate content in lettuce roots and leaves (Gao et al., 2021) and reduced Cu^2+^ toxicity on macrophyte growth (Zhou et al., 2022). PS MPs have been found to reduce the negative effects of As(III) on rice (Dong et al., 2020) and to mitigate the toxic effects of Cu on wheat seedlings (Zong et al., 2021). The underlying mechanisms that enable MPs to mitigate the hazardous effects of these environmental pollutants on plants are I) the reduction in environmental pollutant concentrations in water or soil through the adsorption of environmental pollutants on the MPs surface and II) the inhibition of environmental pollutant uptake into plants by MPs binding on the root surface (Dong et al., 2020; Zong et al., 2021; Zhou et al., 2022). It is possible that the different MPs types (PS vs. PE), MPs sizes (nm vs. μm), MPs exposure concentrations (low vs. high) and treatment durations (hour vs. day), as well as the plant species involved (wheat vs. lettuce), could explain why our study was inconsistent with results in previous research. In summary, Cu(OH)_2_ nanowires greatly affected the vigor of lettuce seeds, the root length of lettuce seedlings and the lettuce antioxidant defence system and MDA content. Expectedly, released Cu^2+^ played a critical role in the toxicity mechanism of Cu(OH)_2_ nanowires in lettuce seedlings. Interestingly, PS MPs (1 mg/L) stimulated lettuce seedling growth. Notably, treatment with PS MPs (1 mg/L) strongly hindered Cu accumulation in lettuce roots and thus neutralized the hazardous effects of the 1 mg/L Cu(OH)_2_ nanowire treatment on lettuce growth. Therefore, PS MPs in the environment could protect lettuce from the hazardous effects of Cu(OH)_2_ nanopesticides and decrease Cu risk in the food chain.

## 4 Conclusions

In this study, the hazardous effects of Cu(OH)_2_ nanowires on lettuce seed germination and seedling growth were confirmed. These effects were highly related to the lettuce antioxidant defence system and MDA content. Released Cu^2+^ was a nonnegligible toxicity mechanism for the hazardous effects of Cu(OH)_2_ nanowires on lettuce. PS MPs (1 mg/L) increased the lettuce seed vigor index and stimulated lettuce seedling growth. Furthermore, the PS MPs treatment (1 mg/L) partially suppressed the hazardous effects of Cu(OH)_2_ nanowires on lettuce growth. This study provides new insights into the toxicity and toxicity mechanisms of Cu(OH)_2_ nanowires and/or PS MPs to plants.

## Data availability statement

The raw data supporting the conclusions of this article will be made available by the authors, without undue reservation.

## Author contributions

GY, data curation, investigation, and writing of the original draft. YS and LY, data curation and investigation. YZ, resources and visualization. WZ, funding acquisition, formal analysis, writing-review, and editing. All authors contributed to the article and approved the submitted version.
